# The Effects of Antipsychotics on Prolactin Levels and Women's Menstruation

**DOI:** 10.1155/2013/502697

**Published:** 2013-12-24

**Authors:** S. I. Bargiota, K. S. Bonotis, I. E. Messinis, N. V. Angelopoulos

**Affiliations:** ^1^Department of Psychiatry, Faculty of Medicine, School of Health Sciences, University of Thessaly, 41110 Larissa, Greece; ^2^Department of Obstetrics and Gynecology, Faculty of Medicine, School of Health Sciences, University of Thessaly, 41110 Larissa, Greece

## Abstract

*Introduction*. Typical and atypical antipsychotic agent is currently used for treatment in the majority of patients with psychotic disorders. The aim of this review is to assess antipsychotic induced hyperprolactinaemia and the following menstrual dysfunction that affects fertility, quality of life, and therapeutic compliance of women. *Method*. For this purpose, Medline, PsychInfo, Cochrane library, and Scopus databases were accessed, with a focus on the publication dates between 1954 and 2012. Research of references was also performed and 78 studies were retrieved and used for the needs of this review. *Results*. A summary of several antipsychotics as well as frequency rates and data on hyperprolactinaemia and menstrual disorders for different agent is presented. *Conclusion*. Diverse prevalence rates of hyperprolactinaemia and menstrual abnormalities have been found about each medication among different studies. Menstruation plays an important role for women, thus, understanding, careful assessment, and management of hyperprolactinaemia could enhance their lives, especially when dealing with women that suffer from a psychotic disorder.

## 1. Introduction

Acute psychotic episodes as well as psychotic relapses are treated effectively with antipsychotic drugs. Most patients with confirmed diagnoses of psychiatric disorders need to undergo antipsychotic drug therapy throughout their whole lives [1, 2]. Typical antipsychotic medications and some of the novel antipsychotics frequently cause an elevation of plasma prolactin levels. Among the several side reactions related with hyperprolactinaemia, are menstrual disorders such as amenorrhea or oligomenorrhea which have not been adequately evaluated. Menstrual dysfunction can be an important source of distress for women, as it influences their libido and fertility [1] and, thus, interferes with their quality of life, a consequence that should be taken into account by clinicians when antipsychotic treatment for each woman is chosen.

This review aims to summarize the effects of antipsychotic agents on prolactin levels and menstruation and investigate the frequency of hyperprolactinaemia and menstrual abnormalities that affect female patients, depending on the selected antipsychotic therapy. It also indicates the need for further research on these adverse effects, the severity of which is not always reported in a clinically meaningful way to experts.

## 2. Background 

### 2.1. How Do Antipsychotics Lead to Hyperprolactinaemia?

A great number of studies have investigated antipsychotic medication and its important effects on human endocrine function. In everyday practice, there are drugs that reduce hypothalamic dopamine secretion and pituitary activation and result in hyperprolactinaemia [3–8].

Conventional antipsychotic agents and some, but not all, of the marketed novel agents, elevate serum prolactin levels via inhibition of dopamine action at D_2_ receptors in the tuberoinfundibular system of hypothalamus, where prolactin secretion is regulated. Specifically, the neurotransmitter dopamine, which acts as the primary prolactin inhibiting factor, is provided to the pituitary gland by the dopaminergic neurons of the periventricular and arcuate nuclei of the medial basal hypothalamus, through the pituitary venus system [1, 9, 10]. Dopamine stimulates D_2_ receptors located on the surface of the lactotroph pituitary cells and provokes a tonic suppression on prolactin secretion. On the other hand, serotonin stimulates prolactin release [5, 9, 11]. In addition, neuropeptides such as thyrotropin releasing hormone TRH, oxytocin, vasoactive intestinal polypeptide VIP, and peptide histidine-methionine, which are under the control of serotonin, promote prolactin (PRL) secretion.

Typical antipsychotic drugs block nonselective dopamine D_2_ receptors in all the regions of the brain. Antipsychotic action that includes reduction of hallucinations, delusions, and other psychotic symptoms is a result of antagonism of dopamine receptors in the limbic system, a fact that raises plasma prolactin levels. By acting to the striatum, classical antipsychotics induce extrapyramidal side effects [5]. Second generation antipsychotics present a higher ratio of serotonin 5HT_2_/dopamine D_2_ receptor binding affinity. Additionally, they have binding affinities for variable neurotransmitter systems, showing selectivity for the mesolimbic than the striatal dopamine system. These agents are called serotonin-dopamine antagonists SDAs, while first generation neuroleptics are potent D_2_ antagonists with low affinity for D_1_ receptor and no significant serotonergic effects [12].

The primary therapeutic target of traditional antipsychotics was the decrease of symptom intensity and the prevention of psychotic recurrence. However, clinicians had to accept hyperprolactinaemia as an implication and a biological marker that came with the drug's efficacy. Data changed in clinical practice after the introduction of novel antipsychotics, which represent an advance in the treatment of psychotic disorders and have a lower tendency to induce hyperprolactinaemia. It has been suggested that the antagonism of 5HT_2_ receptors mitigates the effects of D_2_ receptors inhibition and diminishes extrapyramidal side effects [9].

### 2.2. Side Effects of Hyperprolactinaemia

**Figure 1:**
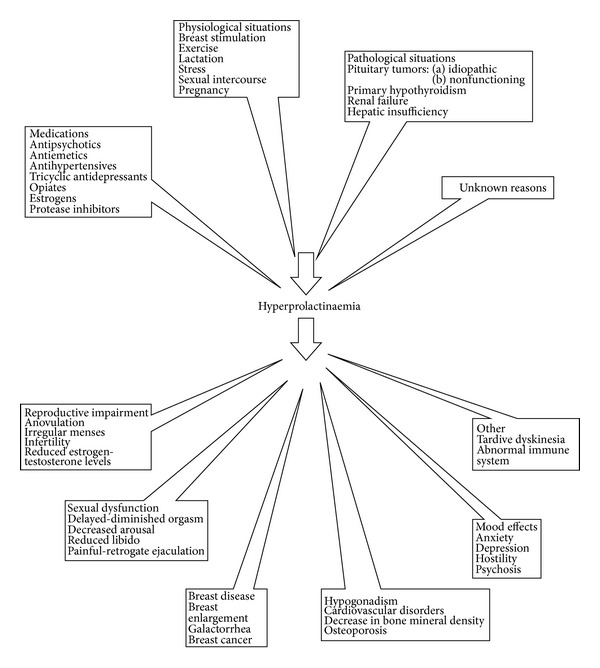
Reasons for hyperprolactinaemia and its side effects.

The majority of clinical adverse effects of hyperprolactinaemia involves the reproductive system and is attributed to prolactin direct relation with several tissues as well as indirect suppression of pulsatile gonadotropin secretion, leading to gonadal dysfunction. Hyperprolactinaemia deregulates systems and processes affected by the pituitary and gonadal hormones (Figure 1).

### 2.3. How Do Antipsychotics Lead to Menstrual Disorders?

**Figure 2:**
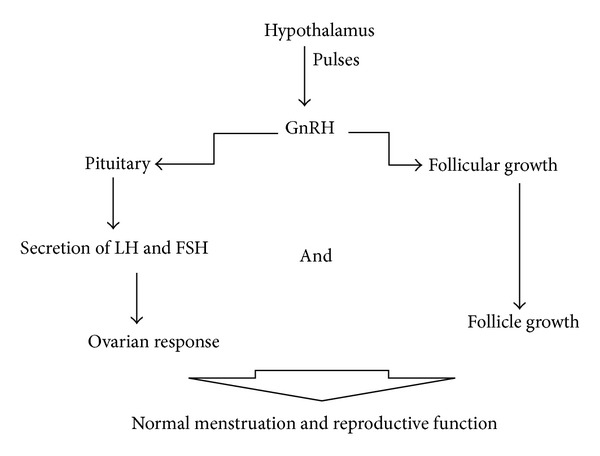
Physiological status of GnRH pulsatile secretion.

When antipsychotics produce hyperprolactinaemia, menstrual abnormalities like anovulation, irregular menses or amenorrhea occur [7–9]. Normally, hypothalamus secrets gonadotropin releasing-hormone—GnRH in a pulsatile manner, resulting in normal follicular growth and normal pituitary secretion of luteinizing hormone—LH and follicle-stimulating hormone—FSH. This action induces normal ovarian response and normal follicle growth and thus, normal menstruation and reproduction (Figure 2).

The great response of prolactin in women of a reproductive age, who are not nursing or pregnant, leads to the inhibition of the normal pulsatile secretion of gonadotropin-releasing hormone (GnRH) of the hypothalamus. These, not so frequent, pulses of GnRH result in regular menses, on the one hand, but impaired follicular growth on the other. Greater impairment of pulsatile GnRH secretion leads to an anovulatory stage with menses being too frequent, too heavy, or infrequent. Further restraining of pulsatile GnRH secretion provokes deficient secretion of LH and FSH, in amounts not adequate to induce a proper ovarian response. That provokes a hypoestrogenized amenorrheic cycle and side reactions of estrogen deficiency—comparable to what occurs during menopause or infertility [13, 14]. Hence, as hyperprolactinaemia is associated with estrogen suppression, the initial prolactin elevation is clinically identified by reproductively related symptoms, primarily in females [9].

### 2.4. Preexisting Menstrual Abnormalities in Women with Psychotic Disorders

Despite the fact that various studies described how antipsychotics lead to menstrual irregularities, sometimes it remained unclear whether menstrual dysfunction was the benign sequale of treatment or it was secondary to the disease. Prior to the introduction of antipsychotic medication, psychotic women were found to have abnormal menses. Amenorrhea is combined with infertility; thus, psychotic illness was supposed to be an indirect, natural contraceptive for female patients [15–17].

Studies in women with schizophrenia proved that they exhibit greater infertility rates compared to healthy females. Some studies support that a high percentage of menstrual irregularity and estrogen deficiency cannot be fully explained by antipsychotic induced prolactin elevation [4]. Another article argued that lifetime psychiatric disorders are associated with the length and regularity of the menstrual cycle only in Caucasians and not in Africans [18]. Aston et al. (2010) supported that it could be stress that leads to hyperprolactinaemia [19]. According to this study, the increase of dopamine levels in psychotic patients could be a feedback mechanism, in order to regulate prolactin elevation, on the one hand, without keeping away the reproductive side reactions, on the other hand. However, there are a lot of studies that suggest that prolactin levels are normal in unmedicated schizophrenic patients [20].

## 3. Materials and Methods

### 3.1. Methods

In order to perform this review numerous studies related to the topic were sought and selected. Most articles were electronically found via databases and citations. Manual research of references was also conducted. The research was carried out using Medline, PsychInfo, Cochrane library, and Scopus and focusing on dates from 1954 to 2010. Studies of each database were extracted and examined. Access to electronic databases was conducted by using the following sequence: #1- menstru∗ OR reproduct∗ OR amenorrhea OR hyperprolactinaemia OR prolactin OR endocrin∗ OR fertility, #2- disorder∗ OR abnormalit∗, #3- #1 AND #2, #4- antipsychotic∗ OR neuroleptic∗, #5- psychot∗ OR psychos∗ OR schizophren∗, #6- #4 AND #5, #7- #3 AND #6. Words with ∗ are root terms (we use the beginning of the word so as more related words can be identified). Selection and examination of the studies were performed and 78 of them were reviewed for the needs of this paper.

### 3.2. Definitions

Prolactin—PRL is a single chain peptide hormone, structurally and evolutionarily homologe to growth hormone GH, as PRL gene on chromosome 6 has 40% similarity to the pituitary GH gene located on chromosome 17 [21]. It was identified as a separate hormone in the early 1970's [2]. PRL receptor (PRL-R), is a transmembrane protein, that is not only located in the breast tissue and in the ovaries but also in peripheral tissues [22]. Pituitary prolactin release is pulsatory and follows a diurnal rhythm. Highest plasma concentration occurs during night sleep and declines during waking periods, reaching a nadir around noon. This circadian rhythm does not depend on sleep but on the circadian pacemaker in the suprachiasmatic nucleus of hypothalamus where prolactin secretion is regulated [9, 10, 14].

The normal levels of prolactin in serum are below 25 *μ*g/L in women and below 20 *μ*g/L in men. 1 *μ*g/L is equivalent to 21,2 mU/L (WHO Standard 84/50).

Hyperprolactinaemia can be defined as an increase in circulating prolactin levels and represents the most common abnormality of pituitary hormones met in clinical practice. There are several reasons responsible for hyperprolactinaemia [10, 11] (Figure 1). Guidelines of Pituitary Society support that PRL values up to 100 *μ*g/L (~2000 mU/L) may be due to psychotropic medications, estrogens, functional causes, or microprolactinomas, while macroadenomas are associated with levels over 250 *μ*g/L (~5000 mU/L) [23].

Menstrual dysfunction was historically defined in association with bleeding patterns (menorrhagia, amenorrhea, oligomenorrhea, polymenorrhea), but now definitions based on ovarian function (anovulation, luteal deficiency) are also used. Another group of menstrual disorders is defined in terms of pain (dysmenorrhea) and onset of bleeding (premenstrual syndrome) [24]. Amenorrhea describes the complete absence of menses for six months. It may be physiological (prepubertal, pregnancy, or postmenopausal) or pathological (disorder at hypothalamic-pituitary-ovarian axis, at uterus or outflow tract) [25]. Oligomenorrhea refers to infrequent periods (cycle length > 35 days) opposite to very frequent periods of polymenorrhea (cycle length > 21 days) [26].

### 3.3. Antipsychotics That Will Be Used in This Review

The term neuroleptics, introduced by Delay in 1955, is not widely accepted. In this paper, we are going to focus on the traditional antipsychotic drugs haloperidol, chlorpromazine, and flupenthixol that along with some of the atypical antipsychotics like risperidone and amisulpride cause an elevation of prolactin levels and menstrual irregularities. Novel antipsychotic agents like clozapine, paliperidone, olanzapine, quetiapine, aripiprazole, ziprasidone, and zotepine, which do not result in hyperprolactinaemia, are also under the scope of this review. However, the terms “prolactin-sparing” and “prolactin-elevating” that will be used in this review and also describe these drugs are believed to be incomplete, because they may lead clinicians to believe that agents like olanzapine and quetiapine can never induce significant hyperprolactinaemia [27, 28].

## 4. Results

A number of 78 articles were examined and included in our study. Endocrine disorders in women provoke several problems like galactorrhea and menstrual disturbances which are responsible for fertility problems [1, 4].

First, Polishuk and Kulcsar in 1956 [29] reported amenorrhea associated with the use of antipsychotic drugs and then several studies followed to support this belief [30, 31]. Although the exact mechanism still remained unknown, they related it with hyperprolactinaemia attributed to conventional antipsychotics. Ghadirian et al. supported the fact that classical antipsychotics frequently show higher incidence of amenorrhea when compared with placebo. Some researchers estimated the prevalence of menstrual disorders in psychotic females on prolactin raising antipsychotic therapy, around 15–50% [32]. Later, Peuskens et al. (1998) [2] reported that amenorrhea occurred in 22–50% of women treated with antipsychotics. In general, the prevalence of menstrual irregularities and amenorrhea is considered to be between 15% and 97% in women receiving therapy for a psychotic disorder [32, 33]. Scientists reported that amenorrhea develops at serum PRL levels above 60–100 ng/mL [34].

### 4.1. Typical Antipsychotics 

Typical antipsychotics, acting as nonselective antagonists of prolactin receptors, are regarded as the most common medications related to hyperprolactinaemia. They lead to acute and persistent increase of prolactin levels [35]. According to multiple studies, the lowest rate of prevalence for typical agents was 33%–35% and the patients received mainly depot drugs. Intramuscular depot administration keeps prolactin levels high for six months after withdrawal of therapy [27, 36]. Hyperprolactinaemia was noticed in 57% of patients receiving typical antipsychotics, in the study by Wong and Seeman (2007) [37]. Montgomery et al. (2004) found prevalence rates for patients in treatment with traditional antipsychotics to be at 68% [28]. A treatment lasting 3–9 weeks, with mostly traditional antipsychotics, can elevate prolactin levels 10-fold above the baseline, and although chronic continuation of the therapy tends to normalize prolactin due to tolerance, it still remains at high levels [38].

The classical antipsychotic drug *haloperidol* has a high binding affinity for dopamine D_2_ and sigma_1_ receptors but a reduced one for 5HT_2A_ and a_1_ receptors. In studies where rates of prevalence were higher, the dose of haloperidol was also higher [39]. The increase in prolactin levels occurs in a dose dependent manner [6, 28, 40].Spitzer et al. (1998), using fifteen patients and their response of prolactin to haloperidol, showed a rapid increase during the first six to nine days between the levels of 30 mg and 50 mg [41]. This elevation was not influenced by dose and remained below 77 ng/mL during the study. Even low dosage of this agent can cause sustained prolactin elevation. Crawford et al. found hyperprolactinaemia at around 72% of the cases at two weeks and around 60% at six weeks of taking haloperidol [42].

*Chlorpromazine *leads to hyperprolactinaemia in the beginning of treatment, a few hours after the first intramuscular or oral intake and persists throughout the whole therapy project [43].* Flupenthixol *has been reported as a half-atypical antipsychotic and elevates serum prolactin levels 2-3 fold in the first month, but these levels normalize in a few months**'** period.

According to Ghadirian et al. (1982) [31], 91% of female patients treated with traditional agents reported a change in their menstruation. Another study conducted by Nonacs (2000) reported that 17% of women treated with conventional antipsychotics developed menstrual abnormalities [44]. Frequency of menstrual dysfunction was decreased after the introduction of prolactin sparing drugs, in women receiving antipsychotics.

### 4.2. Atypical Antipsychotics

Most atypical antipsychotic medications do not elevate serum prolactin levels, in contrast to risperidone which is the exception and leads to a significant increase of prolactin, to a level similar to older antipsychotics [3, 9].

*Risperidone* is a novel antipsychotic that shows a high affinity with 5HT_2A_, 5HT_7_, *α*_1_, H_1_, D_2_, *α*_2_, and 5HT_2D_ receptors, and although it belongs to atypical agents, it has been found to exceed in hyperprolactinaemia compared to conventional antipsychotic drugs [45]. Risperidone has been found in a number of different datasets to raise prolactin in a more substantial and prolonged way than haloperidol does. A percentage of 72%–100% of the females treated with oral risperidone and 53%–67% treated with long-acting intramuscular injection developed hyperprolactinaemia [1, 14, 45]. Kinon et al. (2003) proved that the prevalence rate among women taking risperidone was 88% in contrast to 47% of those taking conventional drugs [6]. A study by Kleinberg et al. (1999) [45] revealed that risperidone was associated with higher mean values of prolactin than haloperidol. However, others found greater elevation in prolactin levels with typical antipsychotics [46].

Risperidone does not completely cross the blood-brain barrier and as a result, tights longer and heavier with D_2_ receptors in the pituitary rather than the striatum. Prolactin levels rise directly some hours after receiving risperidone, reaching maximum levels after eight weeks and maintaining these high levels for a long period of time. A lot of studies demonstrated a correlation between dose of risperidone and prolactin levels, while others did not [47].

The prevalence of menstrual side effects such as amenorrhea in patients on risperidone is reported to be 1%–10% [48], while others support the incidence of abnormal menstrual manifestations to be about 8%–48% of women on risperidone [6]. Another study suggested that there is no significant correlation between plasma prolactin levels and clinical effects of risperidone. Specifically, they found menstrual disorders in only seven out of twenty-seven females under treatment with risperidone for six weeks which is not a high frequency of menstrual dysfunction, but only a tendency to menstrual irregularity symptoms. Women have been found to have greater elevation in plasma prolactin than men when treated with risperidone [7].

*Clozapine* was the first introduced atypical antipsychotic agent and it leads to a short-lived and slight increase of plasma prolactin which may remain undetected in routine laboratory controls [1, 3, 14]. Clozapine binds weakly to dopamine D_2_ receptor and results in transient and low hyperprolactinaemia. This comes in accordance with researches that reported prevalence of hyperprolactinaemia with clozapine treatment from 0% to 5% [37, 49]. Clozapine can sometimes result in a great elevation of prolactin but this is transient and develops in the first few hours. This drug is supposed to reduce hyperprolactinaemia.

Feldman and Goldberg (2002) reported that there is no association between clozapine induced menstrual irregularities and weight gain [50]. Normal menses have returned to women that switched from typical antipsychotics to clozapine [51]. Further studies need to be conducted, related to clozapine and its effects on menstruation.

*Paliperidone *was introduced to Europe in 2007 and it is the active metabolite of risperidone. This 9-hydroxy-risperidone contributes predominantly to hyperprolactinaemia. There are only a few studies about paliperidone induced hyperprolactinaemia and its clinical relevance [52]. Skopek and Manoj (2010) found the elevation of prolactin to be above the normal limit, which resulted after the discontinuation of medication, in four female patients. Values of paliperidone were almost double of those reported for risperidone [53].

*Olanzapine *is an atypical medication that binds intermediately with D_2_ receptor and more tightly with 5HT_2_, at all doses. Olanzapine, which is widely used in Europe, America and Japan, produced transient and mild prolactin elevation compared to that caused by risperidone and haloperidol. In one study with olanzapine and placebo groups there were differences at 2 weeks of therapy but no significant difference was found with regard to the prevalence of hyperprolactinaemia at 6 weeks [42]. According to Kapur et al. (1998) [54], a dose of olanzapine above 30 mg/day induced hyperprolactinaemia equivalent to the one induced by risperidone due to binding with D_2_ receptor occupancy, while other reports suggest that dopamine D_2_ receptor occupancy of risperidone is lower than olanzapine.

The prevalence rate of hyperprolactinaemia in patients on olanzapine has been found to be 68% [49], 28% [37], 40% [29], and 24% [55]. Levels of prolactin have been found to be higher in patients treated with olanzapine and risperidone in comparison to clozapine. Kinon et al. (2006) proved that 90% of patients that were switched to olanzapine were found to have a 50% reduction in prolactin levels, while none of the patients that stayed on prestudy treatment experienced the same decrease [56].

Furthermore, olanzapine treatment improved reproductive comorbid symptoms. Specifically, two out of three women that switched to olanzapine therapy developed a resolution of menstrual disorders opposite to women with menstrual irregularities and prestudy therapy, who continued to have the problem. Additionally, Sawamura et al. conducted a study among Japanese psychotic patients and confirmed gender differences in olanzapine induced prolactin elevation [35]. Nonacs found that no woman on olanzapine experienced endocrine symptoms [44].

*Quetiapine *binds tightly with 5HT_2A_ and has a lower binding affinity for D_2_ receptors in anterior pituitary than most typical antipsychotics and risperidone, and elevates prolactin levels only occasionally. D_2_ occupancy moves from 64%—two hours after dose—to 0%–27%, when twelve hours have passed. Prevalence rates associated with hyperprolactinaemia have been estimated in different studies. Bushe and Shaw report a rate of 0% [49], Wong and Seeman 14% [37], and Polishuk and Kulcsar 22% [29]. Concerning menstrual irregularities, many studies suggest that altering patients' treatment from risperidone to quetiapine could help resume menstruation [57].

*Aripiprazole *is an atypical antipsychotic that is known to act pharmacologically as a partial agonist of D_2_ and 5HT_1A_ and a full antagonist of 5HT_2A_. This leads to low prolactin elevation compared to traditional drugs. Aripiprazole is associated with <5% rate of hyperprolactinaemia [58]. Aripiprazole has been found to provoke a normalization of prolactin levels and menstrual cycles in a woman previously treated with amisulpride and ziprasidone [59].

Benzamides are considered to belong to atypical agents but they were introduced during the 1960's. *Amisulpride *is a substituted benzamide derivative that is not commercially approved in the USA. Although it causes few extrapyramidal symptoms, it provokes a potent prolactin elevating effect, similar to conventional antipsychotics and risperidone [3]. Hyperprolactinaemia occurs after acute and chronic treatment and even in low doses [60], as amisulpride seems to have higher D_2_ occupancy in the pituitary than in the striatum, because it crosses hard the brain-barrier [61].

Amisulpride is regarded to be the antipsychotic with the maximal tendency to cause hyperprolactinaemia. Paparrigopoulos et al. (2007) found that the prevalence rate of hyperprolactinaemia was 100% and this was observed more in women than in men [62]. Amisulpride increases prolactin levels even in low doses, which means that decreased dose of amisulpride has little result on enhancing hyperprolactinaemia [63].

Amenorrhea develops in about 4% of women treated with amisulpride. Menstrual irregularities after usage of amisulpride were also reported in another study [64], but no frequency rate was estimated. Rajnish and Singh (2008) reported that symptoms ameliorate when switching to a prolactin-sparing drug, while there are still no adequately researched studies investigating the prevalence of menstrual abnormalities related to amisulpride [65].

*Ziprasidone * acts as an agonist of serotonin 5HT_1A_ receptors, resulting in a transient and no-sustained elevation of prolactin. Goff et al. compared ziprasidone with haloperidol and found that ziprasidone was associated only with a transient increase in prolactin levels that returned to normal within the dosing interval [66]. One study tried to value ziprasidone adverse reactions and did not find menstrual abnormalities in contrast with risperidone side effects [67].

*Zotepine* is an atypical agent, considered to cause prolactin elevation in humans after acute or long-term therapy [68, 69]. It is uncertain whether there are any published studies that systematically investigate the prevalence of menstrual disorders (Table 1).

## 5. Discussion

Regular, periodical menstruation represents for women an aspect of normality, an indicator of female fertility, and a way to “clean” their bodies [70–72]. It also marks their femininity and health [25]. According to a research conducted in Brazil [70], menstruation was considered by many women to be a “necessary nuisance” determined by nature as an essential part of their reproductive life. Over the last decade, even more contemporary women from diverse cultural backgrounds use methods like contraceptives to suppress menstruation [26, 73, 74].

Nevertheless, menstruation plays an important role in women's lives and any abnormalities interfere with their fertility and quality of life. Especially concerning psychotic women, menstrual disturbances can also influence their compliance to therapy. Therefore, clinicians should examine all aspects before prescribing any medication.

### 5.1. Assessment of a Patient with Hyperprolactinaemia and Menstrual Disorders

In order to asses a woman with hyperprolactinaemia, the clinician should first discover which treatment with antipsychotics resulted in it. Magnetic Resonance Imaging—MRI is the examination of choice so as to investigate the pathological structure in the hypothalamo-pituitary region. If there is any contraindication for MR imaging, then computed tomography scan with contrast, administered intravenously, is the best option [48]. Clinical screening for hyperprolactinaemia should include a carefully taken medical—drug and clinical—history, a physical examination, blood tests, renal, hepatic, and thyroid function tests, as well as testing of visual fields [10].

However, it is worth mentioning that this assessment is often limited to a few questions about clinical manifestations of hyperprolactinaemia and clinicians underestimate hyperprolactinaemia and its side reactions. Another complicating factor is macroprolactinaemia, where a molecular complex of an immunoglobulin G and prolactin is formed. Macroprolactin is biologically inactive as it is restricted to the vascular system but may lead to asymptomatic, falsely elevated prolactin levels [75].

In order to evaluate amenorrhea, a medical history should be carefully taken so as to know if any genital anomalies, thyroid disorders, weight gain, or loss have been observed. Physical examination should be conducted to check for anatomical causes, as well as urine tests to exclude pregnancy. Menstrual status and history is not always adequately documented. Sometimes, the disturbances in menstruation cannot be apparent in short studies. Women might feel stressed and not comfortable to reveal information about reproductive side effects and clinicians might not have the appropriate scales to find out more information [1]. However, they have to monitor for menstrual disturbances during medication therapy. Furthermore, it should be noted that the validity of self-report assessments of menstrual status in psychotic women is uncertain [17].

### 5.2. Management of Hyperprolactinaemia and Menstrual Disorders

Clinicians should be certain about the severity of symptoms and whether they contributed to hyperprolactinaemia or not. Current antipsychotic therapy can be switched to prolactin sparing agents like olanzapine [76], quetiapine, aripiprazole, or clozapine. If this is not feasible, then other treatments, such as estrogen substitution or dopamine agonists, can be used. With regard to dopamine agonists, attention needs to be paid. Although they reduce hyperprolactinaemia, they also cause aggravated acute psychotic episodes in women. Bromocriptine, cabergoline, quinagolide, and amantadine are some common dopamine agonists. Correcting prolactin levels improves symptoms like amenorrhea and other menstrual abnormalities.

Bromocriptine should be prescribed with attention as it resolves amenorrhea but has been found to cause gastrointestinal implications and hypotension [36]. Cabergoline has been found to reduce and normalize prolactin, improving menstrual side effects without deteriorating psychotic symptoms [48]. Some studies support the introduction of aripiprazole in a combination therapy with other antipsychotics for correction of hyperprolactinaemia, but further research is required [77].

### 5.3. Conclusion

Prevalence of hyperprolactinaemia and menstrual disturbances varies not only among antipsychotic agents but also among different researchers (Table 1). Major deviations are observed in prevalence rates of hyperprolactinaemia that occurs during treatment with olanzapine and quetiapine. Most novel antipsychotic agents cause minimal hyperprolactinaemic action or no hyperprolactinaemia at all, compared with classical neuroleptics and risperidone. The study of medication characteristics and interplays contributes to the understanding, assessment, and management of these situations.

Menstrual disturbances like amenorrhea usually recover after prolactin levels have been normalized. However, they can no longer be regarded as a necessary but rather a troublesome consequence of an effective antipsychotic remedy. More studies need to be conducted related to the usage of dopamine agonists and combination therapies for the treatment of prolactin elevation. Clinicians should take into account menstrual abnormalities when they cure women of reproductive age. New antipsychotic agents should be designed to lead to fewer side reactions and improve the lives of psychiatric patients.

## 6. Limitations of the Study 

Antipsychotic induced hyperprolactinaemia is an interesting and important topic and many authors have worked on this. Thus, our review tried to summarize most of the data related to this topic, but may have failed to include all the sources available in the literature.

## Figures and Tables

**Table 1:** Frequency of antipsychotic induced hyperprolactinaemia and menstrual abnormalities according to different studies.

Antipsychotic agents	Prevalence rates of hyperprolactinaemia	Prevalence rates of menstrual abnormalities
All antipsychotic agents		(i) 15%–50% [32]
		(ii) 22%–50% [78]
		(iii) 15%–97% [33]

All typical antipsychotic agents	(i) 33–35% (depot agents) [28, 36]	
	(ii) 47% [6]	
	(iii) 68% [28]	

Haloperidol	72% (2 weeks therapy)—60% (6 weeks therapy), [42]	(i) 91% [31]
		(ii) 17% [44]

Risperidone	(i) 72%–100% (oral treatment)	(i) 1%–10% (Amenorrhea) [48]
	(ii) 53%–67% (intramuscular injection), [1, 14, 49]	
	(iii) 88% [6]	
	0%–5% [27, 37]	(ii) 8%–48% [6]

Clozapine	(i) Double rates of risperidone [52, 53]	
	(ii) No difference [42]	
Paliperidone	(iii) 68% [49]	
	(iv) 40% [37]	
	(v) 28% [28]	
	(vi) 24% [55]	
		
Olanzapine	(i) 0% [49]	No symptoms [44]
	(ii) 14% [37]	
	(iii) 22% [28]	
		
	<5% [58]	

Quetiapine	100% [62]	

Aripiprazole		0% [59]

Amisulpride		(i) 41% (amenorrhea) [63]
		(ii) No adequate data [65]

Ziprasidone		0% [67]

Zotepine		No adequate data [68]
